# Combating Insects Affecting the Health of Man

**Published:** 1916-11

**Authors:** 


### Combating Insects Affecting the Health of Man.

Continued advances in the work of combating the activities of insects affecting the health of man are reported by the Chief of the Bureau of Entomology of the United States Department of Agriculture in his annual report recently issued. In mosquito investigations in Louisiana a species of mosquito hitherto considered a non-carrier of malarial infection was proved to be a carrier. Studies have been made of malaria and measures are being evolved to meet plantation conditions.

The "starvation" plan, aimed to exterminate the spotted fever tick of the Bitter Root Valley, Montana, was followed during the year with encouraging success. The plan consists of the removal of the domestic hosts of the adult tick from the infested areas. The Bureau also conducted a campaign of extermination against ground squirrels and other rodent hosts of the immature ticks. Examination of the rodents killed showed 40 per cent lower infestation by the tick than during the preceding year.

The report directs attention to the demonstrations of the Bureau specialists that the breeding of flies in manure can be prevented by treating the substance with calcium cyanamid and acid phosphate, which at the same time increase the fertilizing value of the manure.

The Bureau also conducted investigations into methods of lessening fly infestation in packing establishments operated under the Meat Inspection Service of the Department.

---

Dr. R. M. Sterrett, many years associated with the advertising of Antiphlogistine, sends greetings to his many friends, announcing his resignation as Advertising Manager of the Denver Chemical Mfg. Co., effective January 1st.

---

The National Board of Medical Examiners held its first examination from October 16 to 21, in Washington, D. C.

There were thirty-two applicants from seventeen States, representing twenty-four medical schools, and of these sixteen were accepted as having the necessary preliminary and medical qualifications, ten of whom took the examination.

The following men passed: Dr. Harry Sidney Newcomer, Johns Hopkins University; Dr. William White Southard, Johns Hopkins University; Dr. Orlow Chapin Synder, University of Michigan; Dr. Thomas Arthur Johnson, Rush Medical School; Dr. Hjorleifur T. Kristjanson, Rush Medical School.

The second examination will be held in Washington, D. C., June, 1917. Further information may be had by applying to Dr. J. S. Rodman, Secretary, 2106 Walnut Street, Philadelphia, Pa.

---

### Do You Know That

It is dangerous to put anything into the mouth except food and drink?

Sanitary instruction is even more important than sanitary legislation?

A branch of the American Society for the Study and Prevention of Tuberculosis was formed in Dallas last week. The session was held at the city hall. It is the purpose to obtain more complete reports than now are available on the number of cases of tuberculosis in that city. Increased hospital facilities and more public nurses will be sought by the society.

